# 79696 Reversible DNA Hypermethylation of the Interleukin-15 Promoter Induces IL-15 Expression, Drives the Pathogenesis of T-Cell Large Granular Lymphocytic Leukemia (T-LGLL) and Provides a Therapeutic Approach Using 5-Azacitidine

**DOI:** 10.1017/cts.2021.638

**Published:** 2021-03-31

**Authors:** Jonathan E Brammer, Anjali Mishra, Amy Boles, Anthony Mansour, Monique Mathe-Allainmat, Agnes Quemener, Pierluigi Porcu

**Affiliations:** 1 The Ohio State University James Cancer Center; 2 Thomas Jefferson University; 3 City of Hope Cancer Center; 4 Universite de Nantes; 5 Universite de Nantes Erwan Mortier, Universite de Nantes

## Abstract

ABSTRACT IMPACT: This work describes, for the first time, the methylome in patients with T-LGLL, focusing on the IL-15 promoter, and clearly demonstrates that 5-azacytidine decreases IL-15 production leading to T-LGLL cell death. These results form the basis a translational clinical trial in T-LGLL that will begin accrual in 2021 OBJECTIVES/GOALS: T-LGLL is an incurable leukemia with few treatment options driven by overexpression of IL-15. Our objective is to characterize the methylation status of the IL-15 promoter in T-LGLL patients and evaluate the potential use of 5-azacytidine (5-aza) in a translational trial by studying the effect of 5-aza in vitro on IL-15 levels, and the IL-15 promoter. METHODS/STUDY POPULATION: We sorted T-LGLL patient (n=3) and normal donor (ND) samples (n=3) for CD3+/CD8+/CD5-/dim for T-LGLL immunophenotype. We analyzed DNA methylation and gene expression profiling using reduced representation bisulfite and RNA sequencing and determined differential methylation and gene expression using 1-way ANOVA analysis. To determine the functional significance of differential methylation, we evaluated MOTN-1 T-LGLL cell viability in vitro with 5-aza at increasing concentrations. Next, we evaluated IL-15 gene expression in MOTN-1 cells treated with 5-Aza versus MOTN-1 with control using western immunoblot. Finally, we exposed MOTN-1 cells to a novel IL-15 inhibitor, IBI-15, and compared cell viability against MOTN-1 cells exposed to an inactive control. RESULTS/ANTICIPATED RESULTS: There was significant differential methylation (P= 0.0178) and expression (P =0.0059) in T-LGLL patients vs ND. These data revealed significant differential hypermethylation of gene promoters, including an increase in DNA methylation of the IL-15 promoter in T-LGLL cells vs ND. In MOTN-1 cells treated in vitro with 5-Aza at 24 and 48 hours, a dose-dependent decrease in the viability of T-LGLL cells was observed, from 100% to 49.5%, p=0.037. Further, a marked decrease in IL-15 expression was observed at all concentrations of 5-aza compared to control (p=0.0001). Finally, a decrease in cell viability was observed utilizing the IL-15 inhibitor IBI-15 vs control. These results confirm that 5-aza leads to decreased transcription of the IL-15 gene, possibly due to hypomethylation of the IL-15 promoter. DISCUSSION/SIGNIFICANCE OF FINDINGS: Hypermethylation of the IL-15 promoter and subsequent increase in IL-15 is critical to the pathogenesis of T-LGLL. Inhibition of the IL-15 promoter by 5-aza leads to down-regulation of the IL-15 gene transcript, which is sufficient to induce T-LGLL cell death. Based on these results, a phase I trial will be conducted using CC-486 (oral 5-Aza) in T-LGLL.

